# Leak after Whipple Resection: Preventable?

**DOI:** 10.1155/1998/23464

**Published:** 1998-12

**Authors:** Charles J. Yeo

**Affiliations:** The Johns Hopkins Medical Institutions Baltimore Maryland USA


While the safety of endoscopic banding ligation is largely undisputed, there is one major risk of this technique: esophageal perforation during insertion of the overtube. Perforation of the esophagus often occurs as a result of inability to relax the pharyngeal muscle by the patient and forceful insertion of the tube by the endoscopist. The development of multiple banding ligators (Speedband™ and Six-shooter™) in recent years has obviated the use of an overtube and is truly major advancement. Intubation is not more difficult with the endoscope loaded with these multiple band ligators and the procedure time is significantly shortened [7, 8]. Recently, a detachable mini-loop ligator has been developed and its use is currently under investigation.

I believe that endoscopic banding ligation will continue to gain popularity. Future development in this treatment modality should be directed to the prevention of recurrent varices formation, the improvement of multiple banding devices and the study of prophylactic banding ligation for selected cases with high risk of variceal bleeding.

### *References*

[1] Laine, L. and Cook, D. (1995). Endoscopic ligation compared with sclerotherapy for treatment of esophageal varices. A meta-analysis. *Ann. Intern. Med.*, **123**, 280-287.

[2] Sung, J. J. Y., Chung, S. C. S., Yung, M. Y., Lai, C. W., Lau, J. Y. W., Lee, Y. T., Leung, V. K. S., Li, M. K. K. and Li, A. K. C. (1995). Prospective randomized study of the effect of octreotide on rebleeding from esophageal varices after endoscopic ligation. *Lancet*, **346**, 1666-1669.

[3] Baroncini, D., Milandri, G. L., Borioni, D., Piemontese, A., Cennamo, V., Billi, P., Dal Monte, P. P. and D'lmperio, N. A. (1997). A prospective randomized trial of sclerotherapy versus ligation in the elective treatment of bleeding esophageal varices. *Endoscopy*, **29**, 235-240.

[4] Lin, C. Y., Lin, P. W., Tsai, H. M., Lin, X. Z., Chang, T. T. and Shin, J. S. (1994). Influence of para-esophageal venous collaterals on efficacy of endoscopic sclerotherapy for esophageal varices. *Hepatology*, **19**, 602-8.

[5] Leung, V. K. S., Sung, J. J. Y., Ahuja, A. T., Tumala, I. E., Lee, Y. T., Lau, J. Y. W. and Chung, S. C. S. (1997). Large para-esophageal varices on endosonography predict recurrence of esophageal varices and rebleeding. *Gastroenterology*, **112**, 1811-1816.

[6] Laine, L., Stein, G. and Sharma, V. (1996). Randomized comparision of ligation versus ligation plus sclerotherapy in patients with bleeding esophageal varices. *Gastroenterology*, **110**, 529-533.

[7] Saeed, A. Z. (1996). The Saeed Six-shoorter: a prospective study of a new endoscopic multiple rubber band ligator for the treatment of varices. *Endoscopy*, **28**, 559-564

[8] Sackmann, M. and Gerbes, A. L. (1996). Application of a multiple band ligator in active variceal bleeding. *Endoscopy*, **28**, 28-533.

Joseph J. Y. Sung M. D., PhD
Department of Medicine
Prince of Wales Hospital
The Chinese University of Hong Kong
Shatin, Hong Kong
China

### Leak after Whipple Resection: Preventable?

### ABSTRACT

*Howard, J. M. (1997) Pancreatojejunostomy: Leakage is a preventable complication of the whipple resection. Journal of the American College of Surgeons;* **184**, *454-457.*

***Background:*** **Leakage of the pancreaticojejunal anastomosis has been a major complication after pancreaticoduodenectomy (Whipple operation), frequently reported in an incidence of 5 percent to 15 percent. The most widely used techniques of anastomosis have been variations of end-to-end pancreaticojejunostomy. Complicating 152 end-to-end anastomoses, done by me (including 98 for carcinoma of the pancreas or ampulla), were 5 pancreatic anastomotic leaks; the fifth patient died of this complication.**

***Study Design*:** **The death resulting from a pancreatic anastomotic fistula led me to change my technique to an end of the pancreas to side of the jejunum, mucosa-to-mucosa, pancreaticojejunostomy (intubated), a modification of the technique described by Cattell and used since**

**1985 by me in 56 consecutive patients. Patients were monitored for clinical evidence of a pancreatic fistula, including evaluation of amylase content in serum and, in most, in peritoneal drainage. Pancreatography through the exteriorized pancreatic catheter was possible if deemed advisable.**
*Results:* **No pancreatic duct was too small or pancreas too soft to permit effective anastomosis. No clinical evidence developed of a pancreatic fistula, "sentinel bleed," or acute pancreatitis, and no patient was recognized to have a high amylase content in the peripancreatic peritoneal drainage. Results of the pancreatogram were negative in three patients with peripancreatic infections and in one with severe cholestasis.**
*Conclusions:* **Although consensus among surgeons does not exist as to technique of pancreatic anastomosis, the end-to-side, mucosa-to-mucosa pancreatico-jejunostomy, intubated, has proved safer in my experience that end-to-end pancreaticojejunostomy. The experience has led me to believe that the technique may reduce the incidence of this fistula and contribute to making pancreaticojejunal leakage a preventable complication.** ***J. Am. Coll. Surg.*****, 1997; 184, 454–457.**

*Keywords:* Whipple operation, pancreatojejunostomy, pancreatic leak

### PAPER DISCUSSION

The report by John Howard entitled "Pancreatojejunostomy: Leakage is a Preventable Complication of the Whipple Resection" serves as a personal review of 56 consecutive end-to-side mucosa-to-mucosa stented pancreaticojejunostomies performed by Dr. Howard since 1985. Dr. Howard has a superb surgical reputation, has immense experience with pancreatic surgery, and is perhaps best known as the individual on whom the character of Trapper John was based from the long-running hit television comedy, MASH.

Several points deserve mention regarding this paper. First, it is beautifully illustrated with four color plates, which unfortunately are not reproduced well by Xeroxing techniques. I would recommend that anyone interested in reviewing this technique obtain the original paper as published in the *Journal of the American College of Surgeons*. Second, the author notes that he performs the jejunal and biliary reconstructions to a Roux-en-Y segment of jejunum, suggesting to me that he is using an isolated Roux-en-Y segment. Third, in order to minimize manipulation of the completed pancreatic anastomosis, the author performs the choledochojejunostomy first, subsequently performing the pancreaticojejunostomy. Other aspects of this particular technique include oversewing of the transected end of the pancreas to augment hemostasis and minimize leakage of pancreatic juice, the use of a transanastomotic catheter which is brought out through the jejunum and the abdominal side wall, and the anastomosis itself is performed with interrupted 4-0 polyglycolic acid-type sutures (Vicryl or Dexon). In the 56 Whipple procedures performed using this technique, no patient had a clinically recognized pancreatic fistula, no patient had evidence of acute pancreatitis, and there was only one postoperative death. These results are impressive and he should be congratulated. Of course, in the absence of a comparative group, the importance of this particular technique in achieving these outcomes cannot be fully substantiated.

Pancreatic fistula remains a major cause of morbidity and mortality after pancreaticoduodenectomy. The most common techniques for managing the pancreatic remnant involve a pancreatic-enteric anastomosis, usually a pancreaticojejunostomy or pancreaticogastrostomy. Many variations of the pancreaticojejunostomy have been reported including the type of anastomosis (invagination versus duct-to-mucosa) the use of an isolated Roux-en-Y limb, the use of pancreatic duct stenting, the site of jejunum used (end *vs.* side), as well as the use of fibrin glue [1–6]. As with many things in surgery, the lack of agreement regarding the safest technique of pancreaticojejunostomy suggests that there may be no one favored technique. Although limited experiences in dogs have favored a duct-to-mucosa technique over invagination [7], and stenting over no stenting [3], prospective, randomized human studies are unfortunately lacking.

Recently pancreaticogastrostomy has gained favor as a potential means of reducing the incidence of pancreatic fistula following the Whipple procedure [8–10]. A prospective, randomized trial from Johns Hopkins enrolled 145 patients and found no differences in the pancreatic fistula rates when comparing between pancreaticojejunostomy and pancreaticogastrostomy [11]. Of note, a multivariate logistic regression analysis performed as part of this prospective, randomized study revealed that the two factors most highly associated with pancreatic fistula were lower surgical volume, and either ampullary or duodenal disease in the resected pathology specimen. Importantly, there appeared to be a linear relationship between the number of cases performed during the study period by each surgeon, and the pancreatic leak rate, with those surgeons performing the greatest number of pancreatic-enteric anastomoses having the lowest leak rate. This underscores the importance of the adage "Practice makes perfect" and would suggest that an experienced pancreatic surgeon may be expected to have the lowest pancreatic fistula rates independent of the method of pancreatic-enteric reconstruction.

A pharmacologic approach designed to reduce the rate of pancreatic fistula involves the perioperative inhibition of pancreatic exocrine secretion. To date, four randomized controlled multicenter trials have evaluated the use of prophylactic octreotide in patients undergoing pancreatic resection [12–15]. Each of these trials was carried out in Europe, used a dose of octreotide of 100 micrograms every eight hours for seven to eight days, and analyzed over 200 patients. Each trial reported statistically significant decreases in overall morbidity, often not solely related to pancreatic fistula. Each trial also reported that the octreotide group had a lower incidence of pancreatic fistula. In none of the trials, however, was there a significant decrease in overall mortality when comparing between the two groups. Some criticisms have been generated concering these European multicenter trials. The trials involved multiple institutions with varying degrees of surgical expertise in pancreatic resection. These trials included multiple types of pancreatic resections, not just pancreaticoduodenectomies. This is important because the rate of pancreatic fistula is known to be lower following distal pancreatectomy, the Peustow procedure and tumor enucleation, as compared to pancreaticoduodenectomy. The trial by Montorsi *et al.*, is the only trial that specifically noted that the pancreatic fistula rate following pancreaticoduodenectomy was *not* significantly different between the octreotide and the placebo groups (11% versus 15%), important information that suggests that octreotide may not lower the pancreatic fistula rate following the Whipple procedure. Another aspect of these European multicenter trials that might be criticized is the high rate of pancreatic fistula seen in the placebo group, ranging from 19% in the Pederzoli *et al.*, trial to 37% in the Buchler *et al.*, trial. These rates of pancreatic fistula are more than double the current pancreatic fistula rates seen at many institutions. At this point it remains unclear how octreotide can influence the overall morbidity rate and the rate of pancreatic fistula in patients undergoing pancreaticoduodenectomy.

There is no doubt that the rate of pancreatic fistula can be decreased with careful surgical techniques and with increasing surgical experience. At this point it is unclear whether pancreatic ductal stenting, a specific type of pancreatic-enteric anastomosis or the use of octreotide actually will decrease the rates of pancreatic fistula. I must admit that I currently favor an end-to-side pancreaticojejunostomy, performed in two layers, attempting to incorporate the pancreatic duct in the inner layer of the anastomosis. I do not use pancreatic ductal stents, I do not spray the anastomosis with fibrin glue, and I do place small bore closed suction drains close to (but not on) the anastomosis. In our experience, roughly 10% of patients will have low volume amylase-rich fluid draining via the drains. Over 85% of these low volume pancreatic fistulas will heal with conservative management. While pancreatic fistula has not disappeared as a postoperative complication, it is certainly no longer the dreaded and feared complication that it was several decades ago. As additional experience and data are gathered, perhaps one particular technique of pancreatic reanastomosis will assume priority.

### *References*

[1] Funovics, J. M., Zoch, G., Wenzl, E. and Schulz, F. (1987). Progress in reconstruction after resection of the head of the pancreas. *Surg. Gynecol. Obstet.*, **164**, 545-548.

[2] Hiraoka, T., Kanemitsu, K., Tsuji, T. *et al.* (1993). A method for safe pancreaticojejunostomy. *Am. J. Surg.*, **165**, 270-272.

[3] Biehl, T. and Traverso, L. W. (1992). Is stenting necessary for a successful pancreatic anastomosis *Am. J. Surg.*, **163**, 530-532.

[4] Kingsnorth, A. N. (1989). Duct to mucosa isolated Roux loop pancreaticojejunostomy as an improved anastomosis after resection of the pancreas. *Surg. Gynecol. Obstet.*, **169**, 451-453.

[5] Kram, H. B., Clark, S. R., Ocampo, H. P., Yamaguchi, M. A. and Shoemaker, W. C. (1991). Fibrin glue sealing of pancreatic injuries, resections and anastomoses. *Am. J. Surg.*, **161**, 479-482.

[6] Matsumoto, Y., Fujii, H., Miura, K. *et al.* (1992). Successful pancreatojejunal anastomosis for pancreatoduodenectomy. *Surg. Gynecol. Obstet.*, **175**, 555-562.

[7] Greene, B. S., Loubeau, J. M., Peoples, J. B. and Elliott, D. W. (1991). Are pancreatoenteric anastomoses improved by duct-to-mucosa sutures *Am. J. Surg.*, **161**, 45-50.

[8] Delcore, R., Thomas, J. H., Pierce, G. E. and Hermreck, A. S. (1990). Pancreatogastrostomy: A safe drainage procedure after pancreatoduodenectomy. *Surgery*, **108**, 641-643.

[9] Kapur, B. M. L. (1986). Pancreaticogastrostomy in pancreaticoduodenal resection for ampullary carcinoma: Experience with thirty-one cases. *Surgery*, **100**, 489-493.

[10] Mason, G. R. and Freeark, R. J. (1995). Current experience with pancreatogastrostomy. *Am. J. Surg.*, **169**, 217-219.

[11] Yeo, C. J., Cameron, J. L., Maher, M. M. *et al.* (1995). A prospective randomized trial of pancreaticogastrostomy versus pancreaticojejunostomy after pancreaticoduodenectomy. *Ann. Surg.*, **222**, 580-592.

[12] Buchler, M., Friess, H., Klempa, I. *et al.* (1992). Role of octreotide in the prevention of postoperative complications following pancreatic resection. *Am. J. Surg.*, **163**, 125-131.

[13] Pederzoli, P., Bassi, C., Falconi, M. *et al.* (1994). Efficacy of octreotide in the prevention of complications of elective pancreatic surgery. *Brit. J. Surg.*, **81**, 265-269.

[14] Montorsi, M., Zago, M., Mosca, F. *et al.* (1995). Efficacy of octreotide in the prevention of pancreatic fistula after elective pancreatic resections: A prospective, controlled, randomized clinical trial. *Surgery*, **117**, 26-31.

[15] Friess, H., Beger, H. G., Sulkowski, U. *et al.* (1995). Randomized controlled multicenter study of the prevention of complications by octreotide in patients undergoing surgery for chronic pancreatitis. *Brit. J. Surg.*, **82**, 1270-1273.

Charles J. Yeo, MD
Professor of Surgery and Oncology
The Johns Hopkins Medical Institutions
Baltimore, Maryland, USA

### Budd-Chiari Syndrome: Shunt or Transplant?

### ABSTRACT

*Hemming, A. W., Langer, B., Greig, P., Taylor, B. R. Adams, R. and Heathcote, J. (1996) Treatment of Budd-Chiari syndrome with portosystemic shunt or liver transplantation. The American Journal of Surgery;* **171***: 176-181.*

*Background:* **Budd-Chiari syndrome is an uncommon disorder caused by obstruction to hepatic venous outflow, causing varying degrees of hepatic injury depending on the extent, severity, and acuity of the obstruction.**

*Patients and Methods:* **We reviewed the indications for operative intervention and the results of treating 32 patients with Budd-Chiari syndrome seen at Toronto Hospital between 1968 and 1995.**

*Results:* **Twenty-one patients underwent porto-systemic shunt (PSS) and 7 patients underwent liver transplantation (LT) as their initial operative management. Three patients who initially had PSS subsequently required LT. Patients with cirrhosis found on biopsy and preservation of hepatocellular function were treated with PSS and showed**

